# Methylammonium Lead Bromide Perovskite Nano-Crystals Grown in a Poly[styrene-co-(2-(dimethylamino)ethyl Methacrylate)] Matrix Immobilized on Exfoliated Graphene Nano-Sheets

**DOI:** 10.3390/nano12081275

**Published:** 2022-04-08

**Authors:** Anastasios Stergiou, Ioanna K. Sideri, Martha Kafetzi, Anna Ioannou, Raul Arenal, Georgios Mousdis, Stergios Pispas, Nikos Tagmatarchis

**Affiliations:** 1 Theoretical and Physical Chemistry Institute, National Hellenic Research Foundation, 48 Vassileos Constantinou Avenue, 11635 Athens, Greece; isideri@eie.gr (I.K.S.); mkafetzi91@gmail.com (M.K.); aioannou@eie.gr (A.I.); gmousdis@eie.gr (G.M.); pispas@eie.gr (S.P.); tagmatar@eie.gr (N.T.); 2 Laboratorio de Microscopias Avanzadas (LMA), Universidad de Zaragoza, Mariano Esquillor s/n, 50018 Zaragoza, Spain; 3 Instituto de Nanociencia y Materiales de Aragon (INMA), CSIC-U. de Zaragoza, Calle Pedro Cerbuna 12, 50009 Zaragoza, Spain; 4 ARAID Foundation, 50018 Zaragoza, Spain

**Keywords:** perovskite nano-crystals, methylammonium lead bromide, defect passivation, free radical polymerization, graphene functionalization

## Abstract

Development of graphene/perovskite heterostructures mediated by polymeric materials may constitute a robust strategy to resolve the environmental instability of metal halide perovskites and provide barrierless charge transport. Herein, a straightforward approach for the growth of perovskite nano-crystals and their electronic communication with graphene is presented. Methylammonium lead bromide (CH_3_NH_3_PbBr_3_) nano-crystals were grown in a poly[styrene-co-(2-(dimethylamino)ethyl methacrylate)], P[St-co-DMAEMA], bi-functional random co-polymer matrix and non-covalently immobilized on graphene. P[St-co-DMAEMA] was selected as a bi-modal polymer capable to stabilize the perovskite nano-crystals via electrostatic interactions between the tri-alkylamine amine sites of the co-polymer and the A-site vacancies of the perovskite and simultaneously enable Van der Waals attractive interactions between the aromatic arene sites of the co-polymer and the surface of graphene. The newly synthesized CH_3_NH_3_PbBr_3_/co-polymer and graphene/CH_3_NH_3_PbBr_3_/co-polymer ensembles were formed by physical mixing of the components in organic media at room temperature. Complementary characterization by dynamic light scattering, microscopy, and energy-dispersive X-ray spectroscopy revealed the formation of uniform spherical perovskite nano-crystals immobilized on the graphene nano-sheets. Complementary photophysical characterization by UV-Vis absorption, steady-state, and time-resolved fluorescence spectroscopy unveiled the photophysical properties of the CH_3_NH_3_PbBr_3_/co-polymer colloid perovskite solution and verified the electronic communication within the graphene/CH_3_NH_3_PbBr_3_/co-polymer ensembles at the ground and excited states.

## 1. Introduction

In the late 2000s, revisiting luminescent lead halide perovskites as photosensitizers in non-silicon solar cells [1] paved the way for a tremendous development of colloid perovskite nano-crystal materials [2]. Protection of these ionic nanostructures from humidity is crucial for their implementation in practical applications. Graphene nano-sheets possessing different chemical groups have been proposed as nanostructured scaffolds for the growth, stabilization, and properties tuning of perovskite nano-crystals. To note, graphene nano-sheets have excellent charge transport properties and have been widely exploited as charge transport layers in perovskite solar cells [3,4,5,6]. A large effort has been put into the design of tightly interacting graphene/perovskite interfaces. In these paradigms, surface chemical functionalities served as capping and passivating agents interacting with the lattice defects of perovskite nano-crystals [7]. Graphene nano-sheets rich in carboxylic acid sites have been used as templates for the in situ growth of inorganic CsPbX_3_ [8] and organic–inorganic CH_3_NH_3_PbX_3_ [9] perovskites. Notably, the oxygen-containing functionalities were found to impact the growth process, as suggested by theoretical approximations [10], nevertheless, it was experimentally validated that the insertion of oxygen defects in the perovskite nano-crystals may occur [9]. Other surface functionalities, varying from phenylamine [11] and iodine [12] to embedded nitrogen atoms [13] and nitrogen/sulphur co-doping [14] were also found to promote crystal growth. Apart from functionalities embedded on the surface of the nano-sheets, supra-molecular graphene-based hybrid materials may expand the available toolkit. Incorporation of N-oxide derivatives of perylene interacting via Van der Waals interactions with the graphitic surface and via the N-oxide unit with the perovskite nano-crystals has been investigated as an alternative supramolecular approach [15]. In analogous fashion, tailoring of the aromatic site of organic cation of organic–inorganic halide perovskites allowed the immobilization of such nano-crystals on the surface of graphene nano-sheets, also via Van der Waals interactions [16]. It is shown that perovskite nano-crystals interfacing graphene nano-sheets are substantially more stable against hydrolysis [17,18,19,20].

In the context of developing efficiently communicating supramolecular graphene/perovskite interfaces, polymer matrices should be considered valuable candidates. Up to the present, a variety of polymers have been implemented for improving the stability of perovskite nanostructures and tuning their photophysical properties. Methylammonium lead halide perovskites, CH_3_NH_3_PbX_3_ (X = Br, I), the eminent members of the organic–inorganic halide perovskite family, have been studied in a series of homopolymers [21,22,23,24,25], as well as di-block co-polymer matrices [26]. Analogous investigations have been reported for inorganic caesium lead halide, CsPbX_3_, perovskite colloid nano-crystals embedded into homo-polymer [27,28,29,30,31,32,33] and co-polymer matrices [34,35,36,37]. In most cases, the anionic: i.e., carboxylate, cationic: i.e., ammonium, or neutral: i.e., pyridine units of the macromolecules serve as capping agents for the growth and stabilization of perovskite nano-crystals. The latter is of importance in controlling the size/shape, as well as the electronic properties of the nano-crystals, including the density of defects. 

Inevitably, bringing together the flexibility of tailored polymeric chains and the high surface area and conductivity of graphene nano-sheets is of wide interest towards functional materials for (opto)electronic, but also catalytic, applications. Graphene oxide having covalently grafted polyacrylic acid chains appeared beneficial for boosting the environmental stability of in situ grown CsPbX_3_ nano-crystals [38]. In general, graphene/perovskite heterostructures mediated by polymeric materials are scarcely explored. In this work, we employed a hydrophobic poly[styrene-co-(2-(dimethylamino)ethyl methacrylate), P[St-co-DMAEMA], a co-polymer where the aromatic rings of styrene units are capable of interacting with the surface of exfoliated graphene nano-sheets via Van der Waals interactions, while the 2-(dimethylamino)ethyl methacrylate units act as passivating agents for the vacant organic cation sites of the perovskite nano-crystals. The growth of CH_3_NH_3_PbBr_3_ nano-crystals into the polymeric matrix, as well as their immobilization on exfoliated graphene nano-sheets, were evaluated by dynamic light scattering measurements, scanning transmission electron microscopy (STEM) and STEM energy-dispersive X-ray spectroscopy (STEM-EDS). Further, the photophysical properties of the perovskite nano-crystals and their electronic communication with graphene were probed by UV-Vis absorption spectroscopy, steady-state and time-resolved photoluminescence (PL) spectroscopy.

## 2. Materials and Methods

### 2.1. Materials

All used reagents are of the Sigma-Aldrich brand (Merck KGaA, Darmstadt, Germany) and used as received, unless otherwise specified.

### 2.2. Instrumentation

^1^H NMR spectrum was recorded using a Varian V300 MHz spectrometer (Varian Inc., Palo Alto, CA, USA). Ultrasonication was performed with the aid of a Bandelin Sonoplus GM3200 (BANDELIN electronic GmbH & Co. KG, Berlin, Germany) equipped with a KE 76 probe. UV-Vis spectra were recorded on a Perkin-Elmer Lambda 19 UV-Vis-NIR spectrophotometer (PerkinElmer, Waltham, MA, USA). Micro-Raman scattering measurements were performed at room temperature in the backscattering geometry using a RENISHAW in Via Raman microscope (Renishaw, Wotton-under-Edge, UK) equipped with a CCD camera and a Leica microscope (Leica Camera AG, Wetzlar, Germany). A 2400 lines/mm grating (for 514 nm) was used, providing a spectral resolution of ±1 cm^−1^. As an excitation source, the Ar-ion laser (514 nm) was used. Measurements were taken with 10 seconds of exposure time and laser power ~0.3 mW/cm^2^ to prevent overheating and damage to the basal plane. The laser spot was focused on the sample surface using a long working distance 50× (L50) objective lens. Raman spectra were collected on numerous spots on the sample and recorded with a Peltier cooled CCD camera. The data were collected and analysed with Renishaw Wire and Origin software. Steady-state emission spectra were recorded on a Horiba GL3-21 Fluorolog-3 Jobin−Yvon−Spex spectrofluorometer (Horiba, Kyoto, Japan), equipped with a 450-W Xe lamp as the excitation source and a TBX photomultiplier (250−850 nm) as the detector, for photoluminescence (PL) measurements. Data were recorded and collected via the Horiba Fluorescence V3 software (Horiba Ltd., Kyoto, Japan). For the pico-second time-resolved fluorescence spectra, a time-correlated single-photon-counting (TCSPC) method via a Fluorohub single-photon counting controller, a laser diode as an excitation source (NanoLED, 482 nm, pulse duration < 200 ps), and a TBX-PMT detector (250−850 nm) -all by Horiba Ltd., Kyoto, Japan- was applied. Data were recorded and collected with the Data Station software, whereas the lifetimes were determined by the Data Acquisition Software (DAS), all provided by Horiba Scientific, Piscataway, NJ, USA. Samples were studied in solution and dispersion forms. Dynamic light scattering (DLS) measurements were conducted on an ALV/CGS-3 compact goniometer system (ALVGmbH, Hessen, Germany), equipped with an ALV 5000/EPP multi-τ digital correlator with 288 channels and an ALV/LSE-5003 light scattering electronics unit for stepper motor drive and limit switch control. A JDS Uniphase 22 mW He-Ne laser (λ = 632.8 nm) was used as the light source. The scattering intensity and correlation functions were measured at a 90° scattering angle. Correlation functions were collected and analysed using the cumulant method and the CONTIN software (ALV-Correlator Software Version 3.0, ALVGmbH, Hessen, Germany), which provide the apparent hydrodynamic radii distributions by Laplace inversion of the correlation function and with aid of the Stokes–Einstein relationship. Scanning transmission electron microscopy high-angle annular field (STEM-HAADF) imaging and energy-dispersive X-ray spectroscopy (EDS) measurements have been performed using an FEI Titan 80–300 kV transmission electron microscope (TEM) working at 120 kV. This microscope is equipped with a condenser lens Cs corrector (CESCOR Cs-condenser, CEOS Company, Heidelberg, Germany), a high brightness field emission gun (XFEG) and an Oxford X-MaxN 100TLE EDS spectrometer. The convergence semi-angle was 25 mrad and particular care has been taken to avoid electron beam damage [39,40]. The samples were dispersed in isopropanol via an ultrasonic bath and dropped onto copper grids coated with a holey carbon film.

### 2.3. Synthesis of the Random Poly[styrene-co-(2-(dimethylamino)ethyl Methacrylate], P[St-co-DMAEMA], Co-Polymer

The random P[St-co-DMAEMA] co-polymer was synthesized using the free radical polymerization (FRP) reaction. Styrene and DMAEMA monomers were initially purified to remove the t-butylcatechol and hydroquinone methylether stabilizers, respectively. Styrene (3.75 g, 0.036 mol) and DMAEMA (1.25 g, 0.008 mol) monomers, AIBN initiator (0.25 g, 0.00152 mol) and 1,4-dioxane solvent (50 mL) were placed in a 100 mL round-bottom flask. Oxygen was removed by bubbling high-purity nitrogen gas through the reaction mixture for 20 min. Then, the flask was sealed and heated in a pre-heated oil bath (70 °C) for 24 h under stirring. After, the flask was cooled in a refrigerator for 10 min and then opened to the atmosphere to quench the polymerization. The reaction mixture was poured in 10-fold excess of hexane to collect the random co-polymer, which, then, was dried in a vacuum oven at r.t. for 48 h. The M_w_ of the random co-polymer was calculated as high as 8600 g/mol with a Μ_w_/M_n_ = 2.3, via gel permeation chromatography, with THF as the solvent using narrow polystyrene standards for calibration. The percentage mol composition was calculated via the ^1^H NMR spectrum, recorded in CDCl_3_, as 74% styrene and 26% DMAEMA.

### 2.4. Preparation of CH_3_NH_3_Br/PbBr_2_ Solution (Perovskite Precursor)

Methylammonium bromide (112 mg, 1.0 mmol), PbBr_2_ (376 mg, 1.0 mmol), and DMF (5 mL) were placed in a 10 mL glass vial furnishing a perovskite precursor concentration of 200 mM. A portion of this solution was further diluted with DMF to a final concentration of 100 mM.

### 2.5. Preparation of CH_3_NH_3_PbBr_3_/Co-Polymer Ensemble

In a 5 mL toluene solution of P[St-co-DMAEMA] co-polymer (C = 10 mg/mL) under stirring, 20 μL of perovskite precursor solution (100 mM in DMF) were added. The mixture turns orange after mixing indicating the growth of the CH_3_NH_3_PbBr_3_ nano-crystals (0.4 mM) and was left under stirring for 24 h. After that period, the saturated solution was diluted with 5 mL toluene affording a final yellowish homogeneous CH_3_NH_3_PbBr_3_/co-polymer solution (0.2 mM).

### 2.6. Liquid-Assisted Exfoliation of Graphene Nano-Sheets

A mixture of 100 mg graphite flakes (>75%, >150 mesh) in 50 mL chlorosulfonic acid * was sonicated for 8 h. During sonication, the temperature was increased from 30 °C to 52 °C. The resulting black homogenous solution was quenched * carefully (highly exothermic reaction) with distilled water. The mixture was filtered through a PTFE membrane filter (pore size 0.1 μm) and washed with water, methanol, and dichloromethane. The filter cake was re-dispersed in NMP (100 mL) with the aid of bath sonication to give a black suspension. Then, the mixture was tip-sonicated (10% power of 150 W, 20 kHz) for 30 min (temperature was kept below 30 °C with a water-ice bath) and the black suspension formed was left to stand for a week at room temperature. After, 2/3 of the black supernatant was collected, filtered through a PTFE membrane filter (pore size 0.1 μm) and washed with water, methanol, and dichloromethane. * Extreme care should be taken when working with chlorosulfonic acid. It reacts violently with humidity and water releasing hydrogen chloride gas. A well-ventilated hood is needed.

### 2.7. Preparation of Graphene/CH_3_NH_3_PbBr_3_/Co-Polymer Ensemble

Exfoliated graphene (1.1 mg) was added in 10 mL toluene and the mixture was tip-sonicated for 3 h (50% power of 150 W, 20 kHz, pulse 5 sec ON-5 sec OFF) in an ice-water bath keeping the temperature below 25 °C. In a 2 mL portion of the grey homogeneous graphene dispersion in toluene (C = 0.1 mg/mL), 2 mL of the CH_3_NH_3_PbBr_3_/co-polymer solution (0.2 mM in toluene) were added and the mixture was left under stirring at r.t. for 36 h. Then, the graphene/CH_3_NH_3_PbBr_3_/co-polymer ensemble was collected via centrifugation (4400 rpm, 5 min) and washed twice with 5 mL toluene under mild sonication, centrifuged (4400 rpm, 5 min) and dried in a vacuum chamber at r.t. The isolated graphene/CH_3_NH_3_PbBr_3_/co-polymer was redispersed in 5 mL toluene with mild sonication and used for the photophysical characterization.

## 3. Results and Discussion

**Figure 1:**
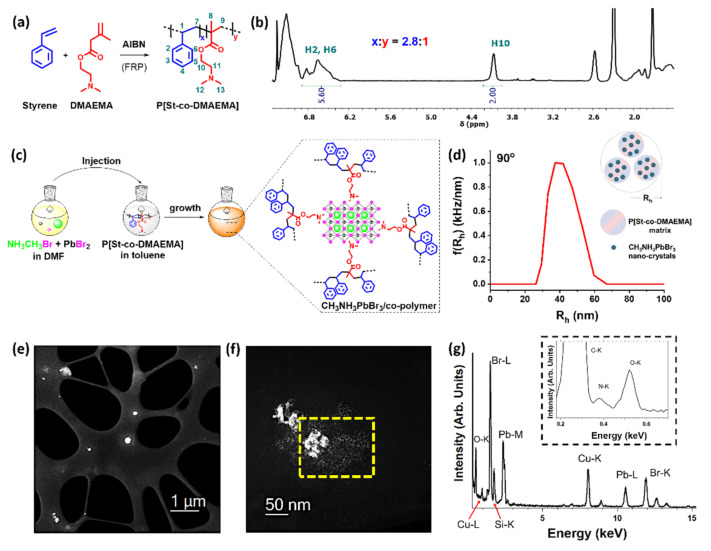
(**a**) Illustration of the synthesis and structure of P[St-co-DMAEMA] co-polymer. (**b**) ^1^H NMR spectrum of the P[St-co-DMAEMA] co-polymer in CDCl_3_ depicting the ratio of the two units. (**c**) Illustration of the synthetic process towards the growth of CH_3_NH_3_PbBr_3_ perovskite nano-crystals in a P[St-co-DMAEMA] co-polymer matrix. (**d**) Hydrodynamic radius distribution by dynamic light scattering at 90° for CH_3_NH_3_PbBr_3_/co-polymer ensembles in toluene. (**e**) Low-magnification and (**f**) high-magnification HAADF-STEM images for the CH_3_NH_3_PbBr_3_/co-polymer ensembles. (**g**) EDS spectrum acquired in the yellow highlighted area of (**f**).

Protecting the perovskite nano-crystals from humidity is of paramount importance to shield these inorganic or organic-inorganic nanostructures from hydrolysis. In this respect, we considered the synthesis of poly[styrene-co-(2-(dimethylamino)ethyl methacrylate)], abbreviated as P[St-co-DMAEMA], co-polymer. Styrene is hydrophobic and its free radical polymerization results in an insulating hydrophobic polymer backbone. The use of DMAEMA monomer for the preparation of the P[St-co-DMAEMA] co-polymer is favourable, since the tri-alkylamine side group is capable to interact with the vacant trimethylammonium sites of CH_3_NH_3_PbBr_3_. Furthermore, the tri-alkylammonium species are more hydrophobic than the methylammonium cation, thus the shielding of ground boundaries from humidity penetration is better achieved. Collectively, P[St-co-DMAEMA] holds meaningful chemical characteristics to promote the growth and protection of perovskite nano-crystals in a hydrophobic environment. Based on a free-radical random polymerization, we prepared the desired P[St-co-DMAEMA] co-polymer (Figure 1a) having a 26% mol percentage of DMAEMA, as calculated via ^1^H NMR (Figure 1b) and a molecular weight of 8600 g/mol, as calculated via gel permeation chromatography (Figure S1). 

A colourless perovskite precursor solution in dimethyl formamide was injected in a toluene solution of the P[St-co-DMAEMA] co-polymer to promote the formation of the CH_3_NH_3_PbBr_3_ nano-crystals at room temperature (Figure 1c). Accordingly, the resulting mixture was quickly coloured orange and displayed bright green photoluminescence under a conventional UV light source, (Figure S2) suggesting the formation of colloid semiconducting perovskite nano-crystals mediated by the co-polymer macromolecules. The growth of uniform CH_3_NH_3_PbBr_3_ embedded into the P[St-co-DMAEMA] matrix was initially accessed by dynamic light scattering measurements, where an average diameter of 74 nm was determined (Figure 1d). Scanning transmission electron microscopy (STEM) imaging and energy-dispersive X-ray spectroscopy (EDS) measurements have been performed to evaluate the morphology of the as-formed fluorescent nanoparticles at the local scale. High-angle annular dark-field (HAADF) STEM imaging of the CH_3_NH_3_PbBr_3_/co-polymer ensembles (Figure 1e–f) revealed nanoscale spherical particles (diameter ~2 nm) with a tendency to form larger aggregates (diameter ~50 nm). Considering the swelling of the co-polymer chains in solution form, the registered diameter value of 74 nm from DLS in solution is comparable to the aggregates (50 nm) observed in the solid-state by STEM imaging. Based on these observations, the colloid suspension of CH_3_NH_3_PbBr_3_/co-polymer ensemble is likely to be dominated by such uniform perovskite nano-crystal aggregates. Further, STEM-EDS spectroscopy (Figure 1g)—recorded in the yellow highlighted area of the HAADF-STEM image of Figure 1f—gave an elemental analysis confirming the presence of CH_3_NH_3_PbBr_3_ nanoparticles. 

**Figure 2:**
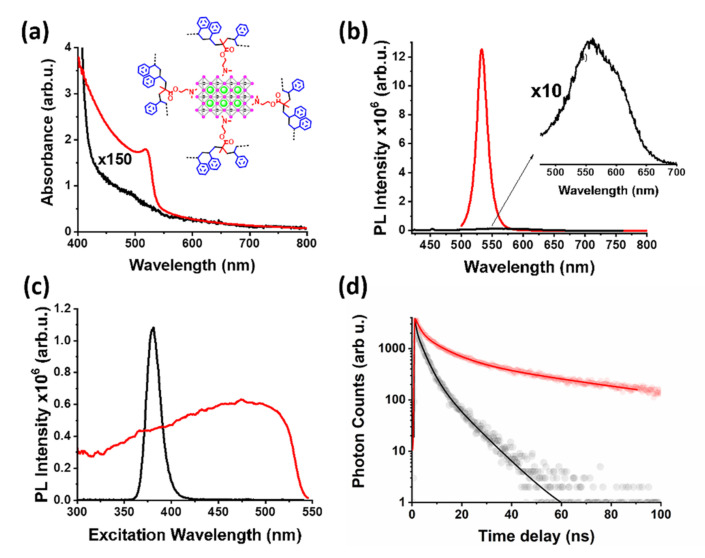
(**a**) UV-Vis, (**b**) steady-state fluorescence emission (exc. 480 nm), (**c**) normalized fluorescence excitation (emi. 533 nm) spectra, and (**d**) time-resolved fluorescence time profiles (exc. 482 nm, emi. 533 nm) for CH_3_NH_3_PbBr_3_/co-polymer ensemble in toluene (red), and the reference perovskite precursor (black). In all cases the final concentration of CH_3_NH_3_Br and PbBr_2_ was 0.2 mM.

The above-mentioned macroscopic observation of colour change, colourless to orange, during the addition of the perovskite precursor solution into the co-polymer solution was further investigated by UV-Vis spectroscopy, where the characteristic excitonic absorption of the colloid CH_3_NH_3_PbBr_3_ perovskite nano-crystals was observed with an absorption maximum at 521 nm (Figure 2a). On the other hand, the solution of the reference perovskite precursor solution without P[St-co-DMAEMA] did not display any excitonic absorption features. Moving forwards, the steady-state fluorescence emission spectrum of CH_3_NH_3_PbBr_3_ was recorded under 480 nm excitation and an intense sharp emission peak centred at 533 nm was observed, indicative of the band-to-band recombination (Figure 2b). Further, the excitation spectrum revealed the broad range of excitation wavelengths resulting from the band-to-band transition (Figure 2c), also the characteristic photophysical property of halide perovskite materials. In contrast, the perovskite precursor in the absence of the co-polymer displayed the weak fluorescence originating solely from the Pb^2+^ cations, as well as a narrow excitation spectrum indicative of the light harvesting properties of free lead cations. The photoluminescence lifetime of the photoexcited bright fluorescent CH_3_NH_3_PbBr_3_/co-polymer ensembles was investigated with the aid of time-resolved fluorescence spectroscopy. The time-resolved PL profile of the reference perovskite precursor solution (Figure 2d, black line) was best fitted with two exponential components t_1_ = 2.1 ns (51%) and t_2_ =7.8 ns (49%). Lead cations are dominating the photophysical properties of the reference sample, as discussed earlier, and the calculated short-lived components are not indicative of perovskite nano-crystals. Injecting the perovskite precursor solution into the co-polymer solution, the growth of CH_3_NH_3_PbBr_3_ within the P[St-co-DMAEMA] co-polymer matrix in toluene resulted in an average lifetime of ~43 ns (Figure 2d, red line). Perovskites commonly exhibit PL time profiles with two distinct mechanisms, a short-lived radiative recombination due to crystal imperfections and a long-lived band-to-band radiative recombination. The PL time profile of the CH_3_NH_3_PbBr_3_/co-polymer ensemble (Figure 1d, red line) was best fitted with two exponential components t_1_ = 9.6 ns (33%) and t_2_ = 60 ns (67%). The long-lived component (60 ns) dominated the PL time profile of the CH_3_NH_3_PbBr_3_/co-polymer ensemble and is attributed to the band-to-band radiative recombination. Further, the short-lived component (9.6 ns) suggests the presence of crystal imperfections, most of which reside at the surface. The tri-alkylammonium units of the co-polymer are capable to populate the vacant cation sites at the surface and therefore prolong the charge separation via passivation of surface charge traps. In summary, the photophysical evaluation validated the growth and stabilization of fluorescent perovskite nanostructures within a hydrophobic co-polymer matrix.

The described approach is evidently a straightforward preparation route towards bright fluorescent CH_3_NH_3_PbBr_3_ nano-crystals grown in a hydrophobic polymer matrix at room temperature, via simple mixing of the perovskite precursor and the bi-functional co-polymer. Taking advantage of the phenyl groups of the polymer matrix we studied the non-covalent immobilization of CH_3_NH_3_PbBr_3_/co-polymer ensemble on exfoliated graphene nano-sheets and more specifically the microscale morphology of graphene/CH_3_NH_3_PbBr_3_/co-polymer ensembles and the electronic communication of the two individual components at the ground and excited states. In this regard, we prepared few-layered graphene nano-sheets via exfoliation in the liquid phase bearing diminutive defects. An undisrupted graphitic network is an ideal candidate for the development of extended Van der Waals interactions with the benzene rings of the polymeric matrix hosting the semiconducting nano-crystals. Additionally, preserving the chemical integrity of the exfoliated nano-sheets is essential to fully exploit the charge transport properties of graphene. To this, graphite powder was initially delaminated by chlorosulfonic acid, followed by ultrasonication in N-methylpyrolidone [41]. The quality of the isolated nano-sheets was screened by Raman spectroscopy, revealing negligible increment of the D band intensity, manifesting diminutive chemical and structural defects, accompanied by a symmetrical 2D band indicating few-layered (<10 atom-thick layers) graphene nano-sheets (Figure S3).

**Figure 3:**
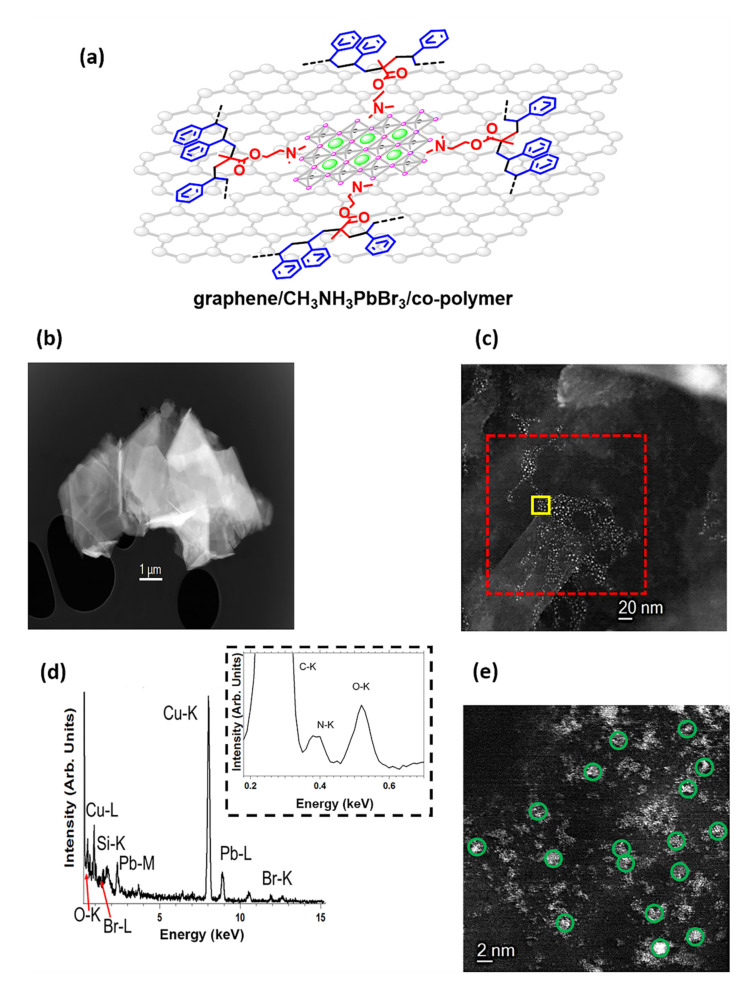
(**a**) Schematic illustration of the graphene/CH_3_NH_3_PbBr_3_/co-polymer ensemble. (**b**) Low-magnification and (**c**) high-magnification HAADF-STEM images for the graphene/CH_3_NH_3_PbBr_3_/co-polymer ensembles. (**d**) EDS spectrum acquired in the red dashed highlighted region of (**c**). (**e**) High-magnification HAADF-STEM image acquired in the yellow highlighted area of (**c**). Green circles represent some of the immobilized nano-crystals.

Hybrid graphene/CH_3_NH_3_PbBr_3_/co-polymer ensembles (Figure 3a) were synthesized by simple mixing of the two components in toluene at room temperature and washing away the free CH_3_NH_3_PbBr_3_ nano-crystals. Microscopic characterization of the as-prepared graphene/CH_3_NH_3_PbBr_3_/co-polymer ensembles via HAADF-STEM confirmed graphene flakes covered by uniformly distributed perovskite nano-crystals (Figure 3b,c). The elemental analysis of the immobilized nanoparticles on the graphitic surface via the acquired EDS spectrum suggested the presence of CH_3_NH_3_PbBr_3_ perovskite nano-crystals (Figure 3d). Interestingly, the CH_3_NH_3_PbBr_3_ nano-crystals within the graphene/CH_3_NH_3_PbBr_3_/co-polymer ensembles are not showing a tendency to aggregate and are well-dispersed on the graphitic surface, as evidenced by HAADF-STEM micrograph (Figure 3e). This observation demonstrates improved morphology for the graphene/perovskite interfaces contrasting the formation of large aggregates in the absence of the graphitic material.

**Figure 4:**
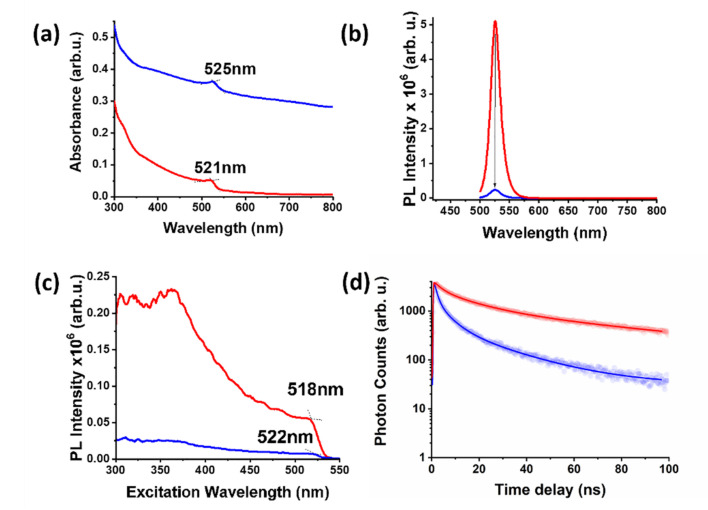
(**a**) UV-Vis, (**b**) steady-state fluorescence emission (exc. 480 nm), (**c**) fluorescence excitation (emi. 533 nm), and (**d**) time-resolved fluorescence emission (exc. 482 nm, emi. 533 nm) spectra for graphene/CH_3_NH_3_PbBr_3_/co-polymer (blue) and a reference equally absorbing CH_3_NH_3_PbBr_3_/co-polymer ensemble (red), in toluene.

The UV-Vis spectrum of purified graphene/CH_3_NH_3_PbBr_3_/co-polymer ensembles displayed the characteristic continuous absorption of graphene and the excitonic absorption peak of the immobilized perovskite nano-crystals. Accumulation of CH_3_NH_3_PbBr_3_/co-polymer ensembles on the two-dimensional graphitic surface redshifted their excitonic absorption to 525 nm, i.e., 4 nm redshift, as compared to an equally absorbing solution of free CH_3_NH_3_PbBr_3_/co-polymer ensembles used as reference (Figure 4a). This redshift is possibly related to ground charge transfer within the graphene/CH_3_NH_3_PbBr_3_/co-polymer ensemble, namely due to electronic communication between the perovskite and the nanosheets. Under photoexcitation at 480 nm, the emission spectrum of graphene/CH_3_NH_3_PbBr_3_/co-polymer ensemble demonstrated dramatic quenching of the fluorescence emission originating from the immobilized CH_3_NH_3_PbBr_3_/co-polymer ensembles (Figure 4b). The latter indicates the intra-ensemble electronic communication between the immobilized perovskite and graphene at the excited states. Recording the excitation spectra of graphene/CH_3_NH_3_PbBr_3_/co-polymer we noticed a 4 nm redshift, as compared to equally absorbing free CH_3_NH_3_PbBr_3_/co-polymer in analogous fashion to the observation from the UV-Vis spectrum (Figure 4c). The observed redshift in the UV-Vis absorbance and PL excitation spectra is possibly originating from charge transfer between the perovskite and the nano-sheets. In order to unveil the fluorescence quenching dynamics within the photoexcited graphene/CH_3_NH_3_PbBr_3_/co-polymer we performed a time-resolved fluorescence spectroscopy study. Under photoexcitation at 482 nm and monitoring the fluorescence emission at 533 nm, we recorded the time-dependent PL profile for the graphene/CH_3_NH_3_PbBr_3_/co-polymer ensembles and the reference CH_3_NH_3_PbBr_3_/co-polymer. The average fluorescence lifetime of the immobilized perovskite on the graphene nano-sheets was found to be ~14 ns (43 ns for the reference CH_3_NH_3_PbBr_3_/co-polymer), manifesting a dynamic quenching mechanism (Figure 4d). More specifically, the PL time profile of the graphene/CH_3_NH_3_PbBr_3_/co-polymer ensembles was best fitted with two exponential components with t_1_ = 5.4 ns (47%) and t_2_ = 23 ns (53%). Both components are faster than those registered for free CH_3_NH_3_PbBr_3_/co-polymer ensembles (9.6 and 60 ns) suggesting that immobilization of the nano-crystals on the graphitic surface is likely to promote faster deactivation of the photoexcited perovskite nanostructures. Further, we concluded that the observed PL quenching is not an internal-filter effect due to the absorbance of graphene nano-sheets. All in all, it was demonstrated that tuning of the graphene/perovskite interface with the aid of the bi-functional P[St-co-DMAEMA] co-polymer resulted in an efficient intercomponent electronic communication at the ground and excited states, thus the co-polymer served not only as a matrix to grow the perovskite nano-crystals but also as an agent for the cohesion of the graphene/perovskite ensemble.

## 4. Conclusions

We described a preparation route towards fluorescent perovskite nano-crystals embedded in a bi-functional P[St-co-DMAEMA] polymer matrix and immobilized on graphene nano-sheets by simple mixing at room temperature. The tri-alkylamine groups of the co-polymer enabled the stabilization of the CH_3_NH_3_PbBr_3_ perovskite nano-crystals into the hydrophobic polymer matrix, while the benzene units of the co-polymer allowed the immobilization of the colloid perovskite nano-crystals on the surface of exfoliated graphene nano-sheets having negligible defects. Scanning transmission electron microscopy imaging and energy-dispersive X-ray spectroscopy verified the formation of CH_3_NH_3_PbBr_3_ in the form of uniform nanoparticles and highlighted the beneficial impact of the bi-functional nature of the co-polymer in perovskite growth and immobilization. CH_3_NH_3_PbBr_3_/co-polymer ensembles formed stable colloid solutions in toluene, as witnessed by dynamic light scattering, enabling their subsequent immobilization on exfoliated graphene nano-sheets via room-temperature solution-processing. STEM imaging of the hybrid graphene/CH_3_NH_3_PbBr_3_/co-polymer ensembles revealed a uniform distribution of the perovskite nano-crystals on the graphitic surface mediated by Van der Waals interactions. Photophysical examination of the graphene/CH_3_NH_3_PbBr_3_/co-polymer ensembles by UV-Vis absorption, steady-state, and time-resolved photoluminescence spectroscopies provided ample evidence for the growth of the perovskite nano-crystals within the polymer matrix and the electronic communication with the graphene nano-sheets at the ground and excited states. All in all, we believe it is a simple approach assisting the utilization of polymer material design towards graphene/perovskite hybrid materials via solution-processing at room temperature. The scope of applications may vary between photocatalysis, photovoltaics, etc., depending on the perovskite, the co-polymer and the two-dimensional material.

## Data Availability

All data generated and analysed during this study are included in this published article and its Supplementary Materials.

## Supplementary Materials

The following supporting information can be downloaded at: https://www.mdpi.com/article/10.3390/nano12081275/s1, Figure S1: Gel permeation chromatograph of the P[St-co-DMAEMA] co-polymer in THF.; Figure S2: Digital photographs of the perovskite nano-crystals solution.; Figure S3: Raman spectra of pristine graphite (grey) and the isolated exfoliated graphene nano-sheets (black) under 514 nm excitation.
