# Supplementary material for: Methylammonium Lead Bromide Perovskite Nano-Crystals Grown in a Poly[styrene-co-(2-(dimethylamino)ethyl Methacrylate)] Matrix Immobilized on Exfoliated Graphene Nano-Sheets
Source: Nanomaterials (Basel). 2022 Apr 8;12(8):1275. doi: 10.3390/nano12081275 (PMC9032388; doi:10.3390/nano12081275)
Supplement: Supplementary file 1 [file nanomaterials-12-01275-s001.zip › nanomaterials-1639210-supplementary.pdf]

nanomaterials

SUPPORTING INFORMATION

Methylammonium Lead Bromide Perovskite Nano-Crystals Grown in a poly[styrene-co-(2-(dimethylamino)ethyl Methacrylate)] Matrix Immobilized on Exfoliated Graphene Nano-Sheets

Anastasios Stergiou [1,*], Ioanna K. Sideri [1], Martha Kafetzi [1], Anna Ioannou [1,†], Raul Arenal [2,3,4,*], Georgios Mousdis [1], Stergios Pispas [1] and Nikos Tagmatarchis [1]

[1] Theoretical and Physical Chemistry Institute, National Hellenic Research Foundation, 48 Vassileos Constantinou Avenue, 11635 Athens, Greece; isideri@eie.gr (I.K.S.); mkafetzi91@gmail.com (M.K.); aioannou@eie.gr (A.I.); gmousdis@eie.gr (G.M.); pispas@eie.gr (S.P.); tagmatar@eie.gr (N.T.)
[2] Laboratorio de Microscopias Avanzadas (LMA), Universidad de Zaragoza Mariano Esquillor s/n, 50018 Zaragoza, Spain
[3] Instituto de Nanociencia y Materiales de Aragon (INMA), CSIC-U. de Zaragoza, Calle Pedro Cerbuna 12, 50009 Zaragoza, Spain
[4] ARAID Foundation, 50018 Zaragoza, Spain
* Correspondence: astergiou@eie.gr (A.S.); arenal@unizar.es (R.A.)
† Present address: Materials Science Department, School of Natural Sciences, University of Patras, 26504 Patras, Greece.

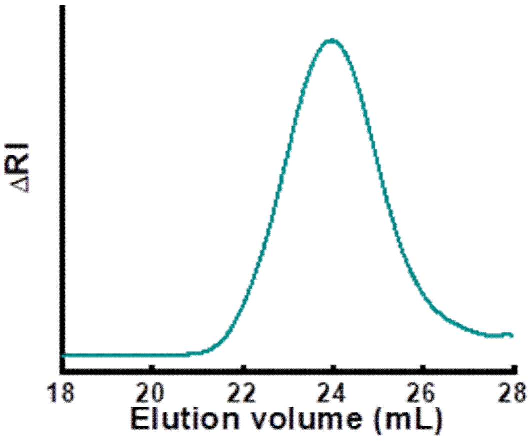

Figure S1. Gel permeation chromatograph of the P[St-co-DMAEMA] copolymer in THF.

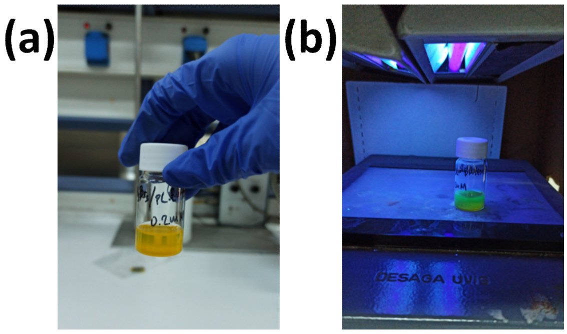

Figure S2. Addition of 20 μL perovskite precursor solution (100 mM in DMF) into a 5 mL toluene solution of P[St-co-DMAEMA] co-polymer (C=10 mg/mL) immediately resulted to a bright orange solution (photograph a, just after mixing) with bright green photoluminescence under a conventional UV light source (photograph b, just after mixing).

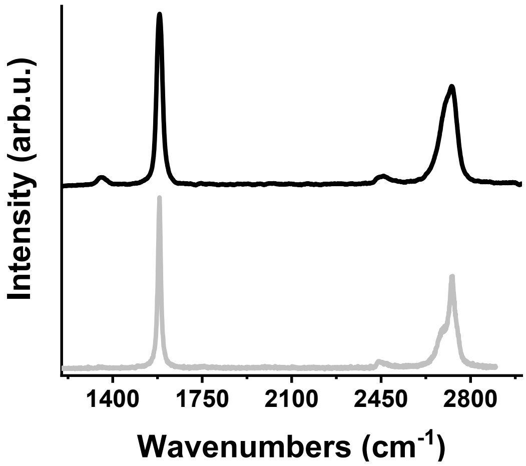

Figure S3. Raman spectra of pristine graphite (grey) and the isolated exfoliated graphene nanosheets (black) under 514 nm excitation.
